# Seven‐Year Longitudinal Respiratory Morbidity in Ohtahara Syndrome: A Case Report Emphasizing Integrated Airway and Seizure Care in a Resource‐Limited Setting

**DOI:** 10.1155/crpe/5989045

**Published:** 2026-07-23

**Authors:** Fadi Abualhommos, Moath Hattab, Masara Samara, Mahmoud Suleiman, Enas Samara, Abdallah Abukhater, Isra Salman

**Affiliations:** ^1^ Faculty of Medicine and Health Sciences, An-Najah National University, Nablus, State of Palestine, najah.edu; ^2^ Department of Medicine, Washington University of Health and Science, Ramallah, State of Palestine, wuhs.edu.bz; ^3^ Pediatrics and Neonatology Department, Tulkarm Governmental Hospital, Tulkarim, State of Palestine

**Keywords:** case report, developmental and epileptic encephalopathy, Ohtahara syndrome, recurrent pneumonia

## Abstract

**Introduction:**

Ohtahara syndrome (OS) features neonatal‐onset tonic spasms with suppression–burst EEG, high early mortality, and evolution to other epileptic encephalopathies. Respiratory complications are frequent yet underdescribed longitudinally.

**Case:**

We followed a female with neonatal‐onset OS and suppression–burst EEG to the age of seven. Despite combination antiseizure therapy (phenobarbital, levetiracetam, vigabatrin, and clonazepam), she had breakthrough events and severe developmental impairment. Between the ages 1 and 7 years, she experienced ≥ 15 documented hospitalizations for recurrent bronchopneumonia, frequently with hypoxemia requiring supplemental oxygen; several episodes were accompanied by seizure exacerbations and clinical aspiration or choking events.

**Management and Outcomes:**

Admissions were managed with supportive respiratory care (oxygen, bronchodilators, and corticosteroids when indicated) and broad‐spectrum antimicrobials alongside ongoing seizure management, enabling survival to school age in a resource‐limited context.

**Conclusions:**

In OS, respiratory complications, often driven by aspiration risk and impaired airway clearance, can become a dominant source of morbidity and should be addressed with the same priority as antiseizure therapy. We highlight practical pillars for long‐term care: proactive infection prevention and early treatment pathways; aspiration‐risk assessment with feeding/swallow support when feasible; airway‐clearance planning with bronchodilator trials during illnesses; judicious corticosteroid use; and caregiver education with explicit thresholds for escalation.

## 1. Introduction

Ohtahara syndrome (OS) is a neonatal‐onset developmental and epileptic encephalopathy (DEE), referring to severe epilepsies in which developmental impairment results from both the underlying brain dysfunction and ongoing epileptic activity. Under the ILAE Classification of Epilepsy Syndromes [1], this electroclinical entity is now encompassed within the broader term early infantile developmental and epileptic encephalopathy (EIDEE); the subsequent evolution to clustered epileptic spasms observed in some patients corresponds to infantile epileptic spasms syndrome (IESS), previously termed West syndrome. Throughout this report, we retain the term “Ohtahara syndrome (OS)” per the journal’s discretion and for historical consistency, while acknowledging current ILAE terminology. It is characterized by frequent tonic spasms and a suppression–burst EEG pattern during wakefulness and sleep. Seizures typically begin in the first weeks to months of life and are often pharmacoresistant. Etiologies include structural brain malformations and pathogenic variants affecting synaptic transmission and interneuron development (e.g., STXBP1 gene mutation). Diagnosis relies on the characteristic EEG pattern and evaluation for structural and genetic etiologies, including neuroimaging and genetic testing when available. In our patient, etiologic testing was limited to targeted *CDKL5* sequencing, which was negative, due to resource limitations. Despite dietary or medical interventions, the prognosis is generally poor, and many infants later evolve to West syndrome or Lennox–Gastaut syndrome. Our patient survived to 7 years but remained severely developmentally impaired with pharmacoresistant seizures and recurrent respiratory hospitalizations; no documented transition to West or Lennox–Gastaut syndrome was available in the records. This context frames our case, which focuses on long‐term respiratory morbidity as a major, under‐appreciated contributor to clinical burden [2–5]. The case adheres to the CARE guidelines for case reports [7].

## 2. Case Presentation

Our patient is a 7‐year‐old female child born at 41 weeks of gestation by elective cesarean section. Her birth weight was 3520 g. Apgar scores were 4 at 1 min and 8 at 5 min. Immediately after birth, she was admitted to the neonatal intensive care unit (NICU) because of meconium aspiration and respiratory distress. She received oxygen via hood at 3.5 L/min, maintaining SpO_2_ of 95%. After 2 days, her vital signs and respiratory distress improved, and she was discharged.

At 6 days of life, she was admitted after her mother noticed cyanosis and abnormal movement of limbs at home. In the hospital, she received a loading dose of phenobarbital as first‐line therapy for suspected neonatal seizures and was admitted for further evaluation. A full septic workup was performed, including venous blood gases (VBGs), liver function tests (LFTs), chest X‐ray, and C‐reactive protein (CRP). VBGs revealed respiratory alkalosis; other laboratories were within normal limits except CRP (9.16 mg/L). CSF analysis and electrolytes were unremarkable except potassium (3.3 mmol/L). It should be noted that no EEG record was retrievable for this admission, likely reflecting the resource‐limited context of our institution. Whether a burst–suppression pattern was already present at this early stage, as would be expected in classic OS, cannot be confirmed from available documentation. The formal electrographic diagnosis (burst–suppression EEG pattern consistent with OS) was established at the 56‐day readmission. This gap underscores a common limitation in resource‐limited settings, where EEG availability and documentation may be inconsistent during acute neonatal presentations. On the following day, she developed central hypotonia with absent Moro and sucking reflexes. Phenobarbital was therefore discontinued, and levetiracetam was initiated. Over the next few days, her convulsions subsided. Two days later, convulsions recurred, and levetiracetam therapy was continued. On Day 18 of admission, CRP was markedly elevated (128 mg/L); therefore, blood cultures were obtained and remained negative. Given concern for possible late‐onset neonatal sepsis, a short empiric course of broad‐spectrum antibiotics (meropenem and vancomycin) was administered, with subsequent clinical improvement.

The patient was discharged at approximately 30 days of age. Neuroimaging was not documented in the available medical records. Head ultrasound, which is relatively inexpensive and accessible, was not recorded as having been performed during the initial neonatal admissions, including at the time of the HIE concern. No brain CT or MRI findings were available for review. This is a significant limitation, as structural neuroimaging is standard in the evaluation of neonatal‐onset epileptic encephalopathies, both to identify causative malformations and to guide prognosis and surgical candidacy. The absence of imaging in our patient likely reflects resource constraints; this prevented confirmation or exclusion of a structural etiology and precluded surgical evaluation. At 56 days of life, she was referred from another hospital with a diagnosis of early neonatal seizures, burst–suppression EEG pattern, hypotonia, poor sucking, and poor motor reflexes. Targeted sequencing of the CDKL5 gene (exons and exon–intron boundaries) was negative for a disease‐causing variant. Broader genetic testing (epilepsy gene panel or exome sequencing) was not available in the treating setting due to limited resources in our country. She was diagnosed with OS. The diagnosis was established on clinical and electrographic grounds, consistent with current diagnostic practice when comprehensive genetic testing is unavailable. Over time, she developed a static motor impairment phenotype consistent with cerebral palsy, likely reflecting early severe brain injury associated with neonatal epileptic encephalopathy. On admission, she was receiving levetiracetam, vigabatrin, and clonazepam. Investigations showed hemoglobin 7.5 g/dL and CRP 50 mg/L. She received a blood transfusion and a short course of meropenem, in addition to continuation of her medications. Then, the patient improved clinically and was discharged home on levetiracetam, vigabatrin, and iron supplementation.

As she grew, her neurological and developmental impairments became more apparent. She had delayed speech and global developmental delay, along with spasticity involving the lower limbs and neck and poor visual function on ophthalmic examination. She had persistent hypotonia since the neonatal period, and over time, she became bedridden and sleepy, consistent with severe neurological sequelae of OS and cerebral palsy.

Her course became more complicated by recurrent respiratory tract infections, often severe enough to require hospitalization. In her second year of life, she was admitted multiple times with fever, productive cough, vomiting, and diarrhea. Initial chest X‐ray was normal, and she was discharged on cefuroxime as a case of hand–foot–mouth disease. Within the same week, she returned with persistent fever and convulsions. This time, chest X‐ray revealed bilateral pneumonia, and she was managed empirically with cephalosporins. Later that year, she had recurrent fever and was managed empirically with cefuroxime.

In her third year, she presented with fever, wheezing, and subcostal retractions. Chest X‐ray was unremarkable, and she was managed with nebulized bronchodilators (ipratropium bromide and salbutamol). Later that year, she was admitted again with bronchopneumonia and treated with salbutamol, ipratropium bromide, and cefuroxime.

In her 4th year, she was admitted with fever, cough, rhinorrhea, and vomiting. Chest X‐ray showed a right sided infiltration, which may indicate pneumonia, and she received bronchodilators and empiric cefuroxime.

In her 5th year, she presented with fever, cough, and rhinorrhea, with oxygen desaturation when not on oxygen support. Chest X‐ray showed bilateral lobar pneumonia. She was managed with oxygen, intravenous antibiotics, bronchodilators, and corticosteroids to reduce airway inflammation and relieve respiratory distress. During this admission, she developed convulsions, requiring levetiracetam loading. Later in the year, she had another similar episode with a normal chest X‐ray, and the same medical regimen was given without antibiotics.

In her 6th year, she had multiple admissions for fever and cough. In one, chest X‐ray showed right‐sided bronchopneumonia, while later that year, she developed bilateral pneumonia, requiring cephalosporins, bronchodilators, and steroids during episodes of significant respiratory distress. In yet another admission, she had a 2‐week history of fever, rhonchi, and transmitted chest sounds, with imaging again showing bilateral pneumonia, treated with empiric cephalosporins.

In her 7th year, she had multiple hospital admissions due to recurrent respiratory distress, aspiration, and seizure exacerbations, mainly described as recurrent generalized convulsive seizures requiring levetiracetam loading. In one admission, she presented with fever, wheezing, and transmitted sound on auscultation, with laboratory findings showing elevated urea, creatinine, sodium, and CRP. In another admission, she developed a choking episode and respiratory difficulty after eating, accompanied by desaturation and decreased air entry bilaterally, requiring oxygen support, nebulized bronchodilators, corticosteroids, and antibiotics. In the next week, she was readmitted with fever and respiratory distress, treated with bronchodilators and steroids. During a subsequent admission within the same month, she had fever and recurrent convulsions requiring levetiracetam loading in addition to her regular antiepileptic regimen, and chest imaging revealed bronchopneumonia.

Microbiological cultures were not consistently obtained, as sputum sampling is rarely feasible in young children; therefore, antimicrobial therapy was initiated empirically.

Overall, from age 1 to 7 years, the patient had ≥ 15 documented hospital admissions for respiratory illness in our records, including recurrent pneumonia (often bilateral/lobar) and bronchopneumonia, frequently associated with hypoxemia requiring supplemental oxygen and escalation to bronchodilators and systemic corticosteroids, with several episodes accompanied by seizure exacerbations. Table 1 summarizes the patient’s course through the 7 years.

**TABLE 1:** Year‐by‐year summary of respiratory admissions and key clinical features (ages 1–7 years).

Year of life	Respiratory admissions	Main presentations	Imaging/key findings	Typical inpatient therapies	Notable associated events
2nd year	≥ 3	Fever, productive cough, GI symptoms	Normal CXR initially; later bilateral pneumonia	Cephalosporins	Convulsions reported with fever in one return visit
3rd year	≥ 2	Fever, wheeze, retractions	One normal CXR; later bronchopneumonia	Nebulized bronchodilators; cefuroxime	—
4th year	≥ 1	URI symptoms + vomiting	Right‐sided infiltration	Bronchodilators + cefuroxime	—
5th year	≥ 2	Fever, cough, rhinorrhea; hypoxemia	Bilateral lobar pneumonia; later similar episode with normal CXR	Oxygen, IV antibiotics, bronchodilators, corticosteroids	Seizure exacerbation requiring levetiracetam loading
6th year	≥ 3	Recurrent fever/cough	Right bronchopneumonia; later bilateral pneumonia (recurrent)	Cephalosporins; bronchodilators; steroids	—
7th year	≥ 4	Recurrent distress; aspiration/choking; fever/wheeze	Bronchopneumonia in one admission; recurrent distress episodes	Oxygen, bronchodilators, corticosteroids, antibiotics	Aspiration episode after eating; recurrent convulsions requiring levetiracetam loading

## 3. Discussion

OS may evolve with age, most commonly transitioning to West syndrome within the first months of life; in one series, 12/16 patients (75%) transitioned, mostly after 3–4 months [8]. This transition is typically associated with replacement of the suppression–burst EEG pattern by hypsarrhythmia and the development of clustered epileptic spasms with ongoing developmental impairment [9]. A smaller proportion of patients may later develop Lennox–Gastaut syndrome, characterized by multiple seizure types and a slow spike–wave EEG pattern, usually before 8 years of age, with peak onset between ages 3 and 5 years [10]. In our patient, however, no follow‐up EEG documentation was available to confirm electroclinical evolution to West syndrome or Lennox–Gastaut syndrome. Her longitudinal course was instead defined clinically by persistent pharmacoresistant seizures, severe global developmental impairment, static motor disability, and recurrent respiratory morbidity through 7 years of age. OS has diverse causes; structural brain abnormalities, especially malformations, are common, and pathogenic variants in genes including KCNQ2, STXBP1, SCN2A, ARX, CDKL5, and SLC25A22 are recognized etiologies, with KCNQ2 mutations accounting for a significant proportion of cases [11].

OS’s neurological impairment is closely linked to structural brain malformations and destructive encephalopathies, which result in central hypotonia, persistent seizure activity with a suppression–burst pattern on EEG regardless of the circadian cycle, and seizures that are extremely intractable. These neurological characteristics cause severe psychomotor retardation, and problems include collapse of the soft tissue structures that maintain a patent airway and affect the swallowing mechanism, leading to airway obstruction and aspiration. Approximately 53% of the reported fatal events were caused by pneumonia or other respiratory illnesses [12]. The severe underlying neurological dysfunction, as well as the limited efficacy of current pharmacologic therapy, contributes significantly to OS’s high morbidity and fatality rates [13, 14].

Respiratory problems are a major cause of morbidity and mortality in epileptic encephalopathies [15]. Khan et al. showed that seizure‐related respiratory episodes may precipitate oxygen desaturation, cardiac arrhythmias, and, in severe cases, sudden unexpected death. Respiratory disturbances can occur during or between seizures and may persist chronically, reflecting disrupted GABA–glutamate balance, impaired central respiratory and autonomic control, and chronic hypoventilation that can progress to hypercapnia and respiratory failure. In OS, pulmonary morbidity is often compounded by aspiration and impaired airway clearance secondary to dysphagia, reduced airway protective reflexes, hypotonia, and immobility; Miller et al. [16] reported diffuse pulmonary changes consistent with chronic aspiration on postmortem examination. Similar respiratory vulnerability has been described across related genetic epileptic encephalopathies, including KCNQ2‐associated disease with apnea, chronic hypoventilation, and recurrent infections [17], and fatal respiratory infections reported in OS with respiratory‐chain Complex I deficiency [18] and BRAT1 mutations [19]. In our patient, recurrent pneumonic admissions alongside choking/aspiration episodes support aspiration and ineffective clearance as key contributors; where available, evaluation should include feeding/swallow assessment, reflux screening, airway anatomy review, and basic immunologic screening when infection frequency appears disproportionate. Table 2 summarizes a practical evaluation and prevention checklist that may guide care in similar settings.

**TABLE 2:** Practical evaluation and prevention checklist for recurrent respiratory morbidity in children with Ohtahara syndrome.

Domain	What to assess	Suggested actions/tests (as available)	When to escalate/refer
Swallow/aspiration	Aspiration risk, dysphagia, choking with feeds	Bedside feeding assessment; consider videofluoroscopic swallow study (VFSS) or fiberoptic endoscopic evaluation of swallowing (FEES) if available	Recurrent choking/aspiration events, recurrent pneumonias, poor weight gain ⟶ speech/swallow team, gastroenterology, multidisciplinary feeding plan
Gastroesophageal reflux	Clinical reflux symptoms contributing to aspiration	Trial of conservative measures (positioning, feeding modifications) ± medical therapy; refer if severe/persistent	Persistent vomiting, feeding intolerance, suspected aspiration, failure to thrive ⟶ gastroenterology ± further testing
Airway/pulmonary anatomy	Structural airway issues, chronic obstruction	Consider ENT/pulmonology review; evaluate for laryngomalacia/tracheomalacia if stridor or atypical wheeze; bronchoscopy if indicated	Stridor, recurrent atelectasis, persistent wheeze, suspected malacia/obstruction ⟶ ENT/pulmonology
Immunology (if feasible)	Disproportionate infection frequency/severity	CBC with differential; serum immunoglobulins; review vaccine responses/records	Unusually frequent/severe infections, poor response to antibiotics, atypical organisms ⟶ immunology referral/workup
Prevention/home plan	Reduce infections and improve early response	Verify influenza + pneumococcal vaccination status; caregiver education; home monitoring thresholds; consider home suction/airway clearance plan if needed	Frequent admissions, baseline hypoxemia, caregiver uncertainty, aspiration risk ⟶ structured home plan + follow‐up schedule

Several case reports in OS have described interventions that may reduce respiratory morbidity. Patel et al. [14] reported an infant with OS and severe stridor who underwent flexible bronchoscopy under ketamine and low‐dose propofol while maintaining spontaneous breathing; bronchoscopy revealed laryngotracheomalacia, and the child’s stridor improved after the procedure and anesthesia despite ongoing respiratory vulnerability. Bael [20] described two patients with OS and recurrent respiratory complications in whom tracheostomy with gastrostomy or gastrojejunostomy feeding was used to reduce aspiration burden and facilitate pulmonary toileting, with fewer respiratory admissions thereafter. Corsano et al. [21] described a newborn with OS who required intubation and mechanical ventilation from birth because of absent spontaneous respiratory activity, underscoring how central respiratory compromise can become in some OS cases. Together, these reports highlight the importance of early identification of airway abnormalities, aspiration prevention through appropriate airway and feeding strategies, and individualized procedural and anesthetic planning to improve respiratory stability in surviving children with OS.

Malik et al. [22] highlighted that OS carries a poor prognosis, due to profound pharmacoresistance where no medical regimen effectively controls seizures. In their study, approximately half of the patients treated with conventional therapy died in early infancy, while survivors remained impaired with persistent, uncontrolled seizures.

Levetiracetam remained part of the long‐term regimen because it was locally available, generally well tolerated, and had been incorporated into the patient’s established seizure‐management strategy. Although ketogenic diet therapy, corticosteroid‐based epilepsy treatment protocols, and epilepsy surgery have been reported in selected DEEs [21], these options were not readily available within the treating healthcare system. Furthermore, no documented focal structural lesion was available to support consideration of epilepsy surgery. The potential benefits of corticosteroid therapy also had to be weighed against the patient’s substantial burden of recurrent respiratory infections.

Conversely, outcomes for surgically treated patients are generally better. Procedures such as focused cortical resection, lobectomy, and hemispherectomy aim to remove or disconnect the epileptogenic cortex. When performed early in life, surgery can prevent seizures, slow neurological decline, and promote developmental recovery. In Malik et al.’s series, most surgically treated newborns achieved seizure freedom or significant improvement, suggesting that early epilepsy surgery may offer better outcomes than medication alone in carefully selected infants [22]. Surgery is generally considered when neuroimaging and EEG identify a focal or lateralized epileptogenic lesion, such as cortical dysplasia, hemimegalencephaly, porencephaly, or another resectable/disconnectable structural abnormality. Therefore, this option requires multidisciplinary epilepsy‐surgery assessment and is not applicable to all newborns with OS.

Our patient’s course was similar to the classical OS phenotype. Seizures began in the neonatal period, EEG demonstrated suppression–burst, and the patient also had early hypotonia and poor suck. Despite the combination of phenobarbital, levetiracetam, vigabatrin, and clonazepam, breakthrough events occurred, consistent with the well‐recognized pharmacoresistance of OS. Although severe neurological dysfunction was the primary driver of hypotonia in this patient, sedating antiseizure medications, particularly phenobarbital in early infancy, may also have worsened tone, feeding ability, and airway protection during parts of the course. The recurrent respiratory burden stood out in our case over the years documented by repeated admissions for pneumonia or bronchopneumonia, hypoxemia requiring supplemental oxygen, and frequent use of bronchodilators, systemic steroids, and broad‐spectrum antibiotics. Repeated empiric antimicrobial treatment reflected recurrent fever, hypoxemia, inflammatory‐marker elevation in some admissions, and radiographic pneumonia or bronchopneumonia in several episodes, although we acknowledge that not all events were microbiologically confirmed and some may have been aspiration‐related rather than proven bacterial infection. Repeated empiric cephalosporin administration reflected local treatment practices and the challenges of managing recurrent respiratory illness in a setting where microbiological confirmation was often unavailable. Nevertheless, prolonged or repeated exposure to broad‐spectrum antibiotics may contribute to antimicrobial resistance, alter respiratory flora, and complicate future treatment decisions. Antimicrobial stewardship and culture‐guided therapy should be prioritized whenever feasible. We acknowledge that some admissions may have reflected aspiration‐related pulmonary inflammation rather than proven bacterial infection. This pattern mirrors published series in which respiratory illness and sudden unexpected death in epilepsy (SUDEP) were the leading proximate causes of death in early infantile epileptic encephalopathies. At the same time, this child’s survival into school age, though accompanied by severe developmental impairment, reflects an ongoing pattern of heterogeneous survivorship due to modern supportive care, which depends as much on respiratory and nutritional support as on seizure control alone. At her current age of 7 years, she has profound global developmental delay across multiple domains: Gross motor function is severely impaired, with bedridden status and spasticity involving the lower limbs and neck; speech and cognitive development are markedly delayed; visual function is poor on ophthalmic assessment; and she remains dependent on caregivers for daily care and feeding. This child was managed in a resource‐limited setting, where access to broader genetic testing, structured swallow evaluation, and advanced respiratory support pathways was constrained, likely affecting both diagnostic precision and long‐term respiratory management.

One critical domain not addressed in this report is palliative care. In neonatal epileptic encephalopathies such as OS/EIDEE, early goals‐of‐care discussions are considered best practice and should be initiated at the time of diagnosis, revisited with each significant clinical change, and reinforced at every hospital admission. These discussions should include the following: (1) the family’s understanding of the diagnosis, prognosis, and expected disease course; (2) their values, beliefs, and cultural context regarding life‐sustaining interventions; (3) explicit discussion of resuscitation status (code status) and acceptable levels of invasive support; and (4) preferences regarding tracheostomy, gastrostomy tube, prolonged mechanical ventilation, and escalation to ICU care [1, 23]. Resource‐limited settings do not exempt clinicians from these obligations; indeed, they make them more urgent, as families may face decisions about interventions that they cannot sustain long‐term or that may impose a significant burden without proportionate benefit.

It is also worth mentioning that a negative CDKL5 exon sequencing result does not exclude other genetic etiologies of OS (e.g., STXBP1, KCNQ2, SCN2A, and ARX). A key limitation is that comprehensive molecular testing was unavailable; therefore, despite negative targeted CDKL5 sequencing, the etiologic diagnosis remained clinic‐electrographic rather than molecularly confirmed [24].

## 4. Conclusion

This 7‐year follow‐up highlights that, in OS, respiratory disease can become the dominant driver of morbidity, occurring repeatedly alongside pharmacoresistant seizures and severe neurodevelopmental impairment. In our patient, recurrent pneumonia/bronchopneumonia with hypoxemia and aspiration events led to frequent hospitalizations and periodic seizure exacerbations, underscoring the need to manage airway and infection risk with the same priority as antiseizure therapy. A practical long‐term approach should include the following: (1) structured infection prevention and early‐treatment pathways; (2) proactive aspiration‐risk identification with feeding support and swallow assessment when feasible; (3) planned airway‐clearance strategies (positioning, physiotherapy, and suctioning as indicated) with bronchodilator trials during acute illnesses; (4) careful, episode‐specific use of corticosteroids; and (5) clear caregiver escalation thresholds and home monitoring plans. Embedding these elements into standardized follow‐up protocols may reduce admissions and improve clinical stability, particularly in resource‐limited settings.

## Author Contributions

Study conception/design: Moath Hattab, Masara Samara, and Isra Salman.

Clinical data acquisition: Moath Hattab, Masara Samara, Mahmoud Suleiman, and Isra Salman.

Literature review: Moath Hattab, Fadi Abualhommos, Mahmoud Suleiman, Enas Samara, and Abdallah Abukhater.

Manuscript drafting: Moath Hattab, Masara Samara, Fadi Abualhommos, Mahmoud Suleiman, Abdallah Abukhater, Enas Samara, and Isra Salman.

Critical revision and supervision: Moath Hattab and Isra Salman.

## Funding

All authors have no source of funding.

## Disclosure

All authors reviewed and approved the final manuscript.

A preprint version of this manuscript has previously been published: Hattab et al. [6].

## Ethics Statement

Our institution does not require ethical approval for case reports.

## Consent

The patient’s legal guardians (parents) provided written informed consent for the publication of this case report. The editor‐in‐chief of this journal can review a copy of the consent on request.

## Conflicts of Interest

The authors declare no conflicts of interest.

## Supporting Information

Additional supporting information can be found online in the Supporting Information section.

## Supporting information


**Supporting Information** CARE checklist for Ohtahara.

## Data Availability

The data supporting the findings of this case report are not publicly available due to patient privacy and consent restrictions. Deidentified information may be made available from the corresponding author upon reasonable request, subject to institutional approval and patient guardian consent.
