# Supplementary material for: Seven‐Year Longitudinal Respiratory Morbidity in Ohtahara Syndrome: A Case Report Emphasizing Integrated Airway and Seizure Care in a Resource‐Limited Setting
Source: Case Rep Pediatr. 2026 Jul 23;2026:5989045. doi: 10.1155/crpe/5989045 (PMC13396428; doi:10.1155/crpe/5989045)
Supplement: Supplementary file 1 — Supporting Information CARE checklist for Ohtahara. [file CRPE-2026-5989045-s001.pdf]

CARE Checklist

Seven-Year Longitudinal Respiratory Morbidity in Ohtahara Syndrome: A Case Report Emphasizing Integrated Airway and Seizure Care in a Resource-Limited Setting

Topic	Item	Checklist item description	Reported on line
Title	1	The diagnosis or intervention of primary focus followed by the words "case report".	Lines 1-2
Key Words	2	2 to 5 key words that identify diagnoses or interventions in this case report, including "case report".	Lines 33-34
Abstract	3a	Introduction: what is unique about this case and what it adds to the scientific literature.	Lines 15-17 and 27-32
Abstract	3b	Main symptoms and/or important clinical findings.	Lines 18-23
Abstract	3c	The main diagnoses, therapeutic interventions, and outcomes.	Lines 18-26
Abstract	3d	Conclusion: the main take-away lesson(s) from this case.	Lines 27-32
Introduction	4	One or two paragraphs summarizing why this case is unique, with references where appropriate.	Lines 35-58
Patient Information	5a	De-identified patient-specific information.	Lines 61-66
Patient Information	5b	Primary concerns and symptoms of the patient.	Lines 67-68 and 113-148
Patient Information	5c	Medical, family, and psychosocial history including relevant genetic information.	Lines 62-66 and 95-103
Patient Information	5d	Relevant past interventions with outcomes.	Lines 68-86, 104-107, and 120-148
Clinical Findings	6	Significant physical examination and important clinical findings.	Lines 79-81, 108-112, and 140-144
Timeline	7	Historical and current information organized as a timeline.	Lines 113-157, especially Table 1 at lines 151-157
Diagnostic Assessment	8a	Diagnostic testing, including physical examination, laboratory testing, imaging, and surveys.	Lines 69-76, 87-100, and 120-148
Diagnostic Assessment	8b	Diagnostic challenges, including access, financial, or cultural barriers.	Lines 73-79, 87-100, 149-150, 269-271, and 283-287
Diagnostic Assessment	8c	Diagnosis, including other diagnoses considered.	Lines 84-85, 95-103, and 115-118
Diagnostic Assessment	8d	Prognosis, where applicable.	Lines 51-55, 217-220, and 272-282
Therapeutic Intervention	9a	Types of therapeutic intervention, such as pharmacologic, surgical, preventive, or self-care.	Lines 24-26, 68-86, 104-107, 120-148, and 221-228
Therapeutic Intervention	9b	Administration of therapeutic intervention, such as dosage, strength, and duration.	Lines 65, 68, 80-86, 104-107, and 120-148
Therapeutic Intervention	9c	Changes in therapeutic intervention, with rationale.	Lines 79-85 and 221-228
Follow-up and Outcomes	10a	Clinician- and patient-assessed outcomes, if available.	Lines 86, 106-107, 151-155, 239-248, and 264-268 (patient-assessed outcomes not reported/limited by age and impairment)
Follow-up and Outcomes	10b	Important follow-up diagnostic and other test results.	Lines 120-148, 151-157, and 165-167
Follow-up and Outcomes	10c	Intervention adherence and tolerability, including how this was assessed.	Lines 221-223 and 244-246
Follow-up and Outcomes	10d	Adverse and unanticipated events.	Lines 127-148, 151-155, and 244-246
Discussion	11a	Scientific discussion of the strengths and limitations associated with this case report.	Lines 269-271 and 283-287
Discussion	11b	Discussion of the relevant medical literature with references.	Lines 159-220 and 303-367
Discussion	11c	Scientific rationale for conclusions, including assessment of possible causes.	Lines 173-199, 246-259, and 289-301
Discussion	11d	Primary take-away lessons of this case report in a one-paragraph conclusion, without references.	Lines 288-301
Patient Perspective	12	The patient should share their perspective in one to two paragraphs on the treatment(s) received.	Not reported.
Informed Consent	13	Did the patient give informed consent? Please provide if requested.	Lines 375-377
